# Supplementary material for: New-Generation Heterocyclic Bis-Pentamethinium Salts as Potential Cytostatic Drugs with Dual IL-6R and Mitochondria-Targeting Activity
Source: Pharmaceutics. 2022 Aug 17;14(8):1712. doi: 10.3390/pharmaceutics14081712 (PMC9416741; doi:10.3390/pharmaceutics14081712)
Supplement: Supplementary file 1 [file pharmaceutics-14-01712-s001.zip › pharmaceutics-1825418-supplementary.pdf]

New-Generation Heterocyclic Bis-Pentamethinium Salts as Potential Cytostatic Drugs with Dual IL-6R and Mitochondria-Targeting Activity

Veronika Talianová [1,2], Zdeněk Kejík [1,2], Robert Kaplánek [1,2], Kateřina Veselá [1,2], Nikita Abramenko [1,2], Lukáš Lacina [1,3,4], Karolína Strnadová [1,3], Barbora Dvořánková [1,3], Pavel Martásek [2], Michal Masařík [1,2,5], Magdalena Houdová Megová [6], Petr Bušek [6,*], Jana Křížová [2], Lucie Zdražilová [2], Hana Hansíková [2], Erik Vlčák [7], Vlada Filimonenko [7], Aleksi Šedo [6], Karel Smetana Jr. [1,3], Milan Jakubek [1,2,*]

Content:

Figure S1: Absorption spectra of Bis-PMS **5** and **6** in four different solvents
Figure S2: Excitation and emission spectra of Bis-PMS **5** in four different solvents
Figure S3: Excitation and emission spectra of Bis-PMS **6** in four different solvents
Figure S3: Normalized bleaching curves of Bis-PMS **5** and **6**
Figure **S4**: Absorption spectra of Bis-PMS **5** after incubation points in EMEM with and without MeOH
Figure S5: Absorption spectra of Bis-PMS **6** after incubation points in EMEM with and without MeOH
Figure S6: Normalized bleaching curves of Bis-PMS **5** and **6**
Figure S7: Interaction of mono penthamethinium salts with a 3D model of IL-6R
Figure S8: Interaction of monopentamethium salts with the residues of homology model of IL-6R
Figure S9: Effect of bis-pentamethinum salts **5** and **6** on the IL-6 signaling cascade
Figure S10: Cytotoxity of Bis-PMS **5**
Figure S11: Cytotoxity of Bis-PMS **6**
Figure S12: Cytotoxity of Bis-PMS **5** and **6**
Figure S13: In vitro localization of Bis-PMS **5**
Figure S14: In vitro localization of Bis-PMS **6**
Figure S15: 2-D scatter plots of fluorescent probes Bis-PMS **5** and **6** versus MitoTracker
Figure S16: Influence of Bis-PMS **5** and **6** on the mitochondrial metabolism and respiration
Figure S17: Influence of Bis-PMS **5** and **6** on the glycolytic function
Figure S18: Measurements of respiration (OCR) and glycolysis (ECAR) of melanoma cells
Figure S19: Influence of Bis-PMS **5** on mitochondrial morphology of A2058 cells
Figure S20: Influence of Bis-PMS **6** on mitochondrial morphology of A2058 cells
Figure S21: Mitochondrial morphology of HFP4
Figure S22: Influence of Bis-PMS **5** on mitochondrial morphology of HFP4 cells
Figure 23: Influence of Bis-PMS **6** on mitochondrial morphology of HFP4 cells
Table S1: Excitation and emission maxima of Bis-pentamethinium salts **5** and **6**
Table S2: Interaction mode of Bis-pentamethinium salts **5** and **6** with amino acid residue of IL-6R
Table S3: Cytotoxicity of Bis-PMS **5** and **6** in normal and cancer **cells**

Photophysical characteristics of bis-pentamethinium salts 5 and 6

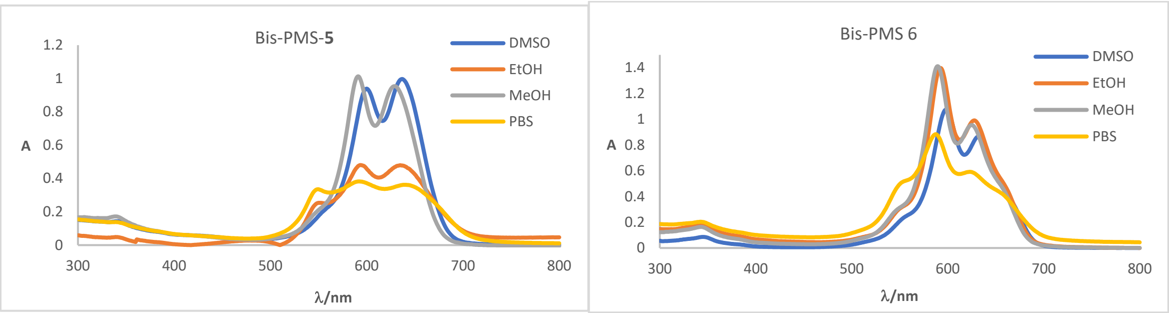

Figure S1: Absorption spectra of Bis-PMS **5** and **6** (**5.5** μM) in four different solvents(DMSO, EtOH, MeOH and PBS).

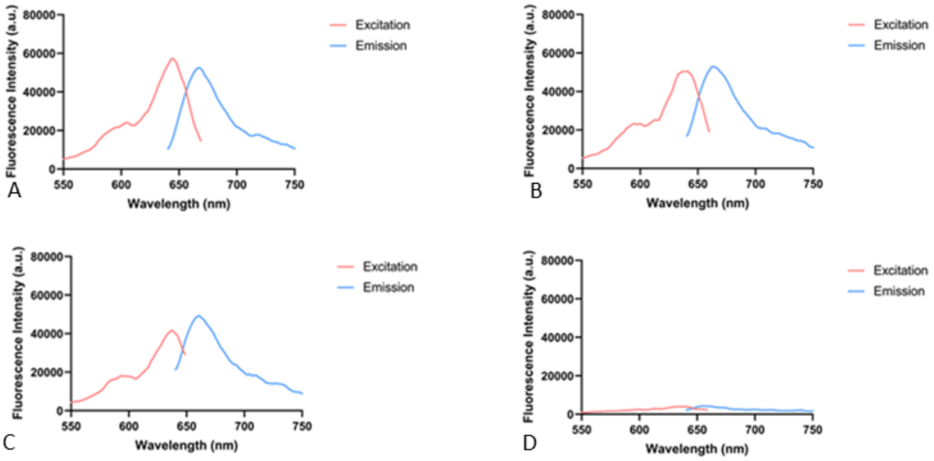

Figure S2: Excitation and emission spectra of Bis-PMS **5** (0.5 μM) in four different solvents. (**A**) DMSO, (**B**) EtOH, (**C**) MeOH, (**D**) PBS.

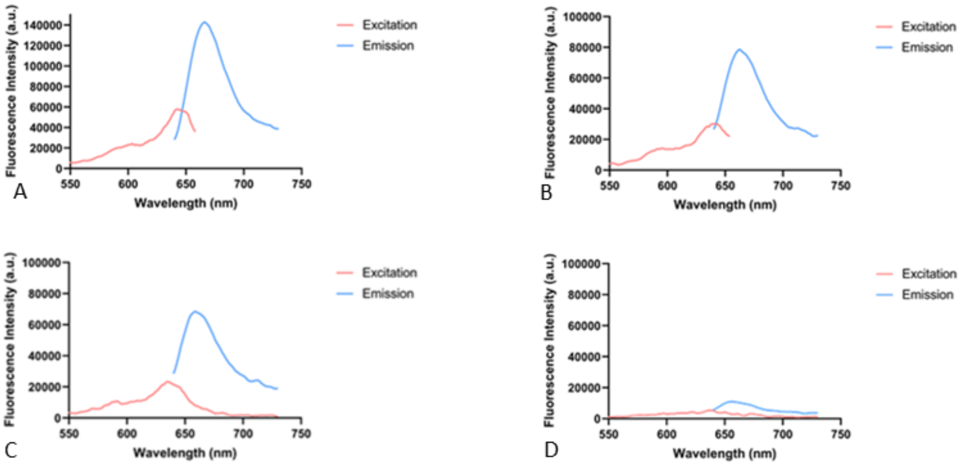

Figure S3: Excitation and emission spectra of Bis-PMS **6** (0.5 μM) in four different solvents (**A**) DMSO, (**B**) EtOH, (**C**) MeOH and (**D**) PBS.

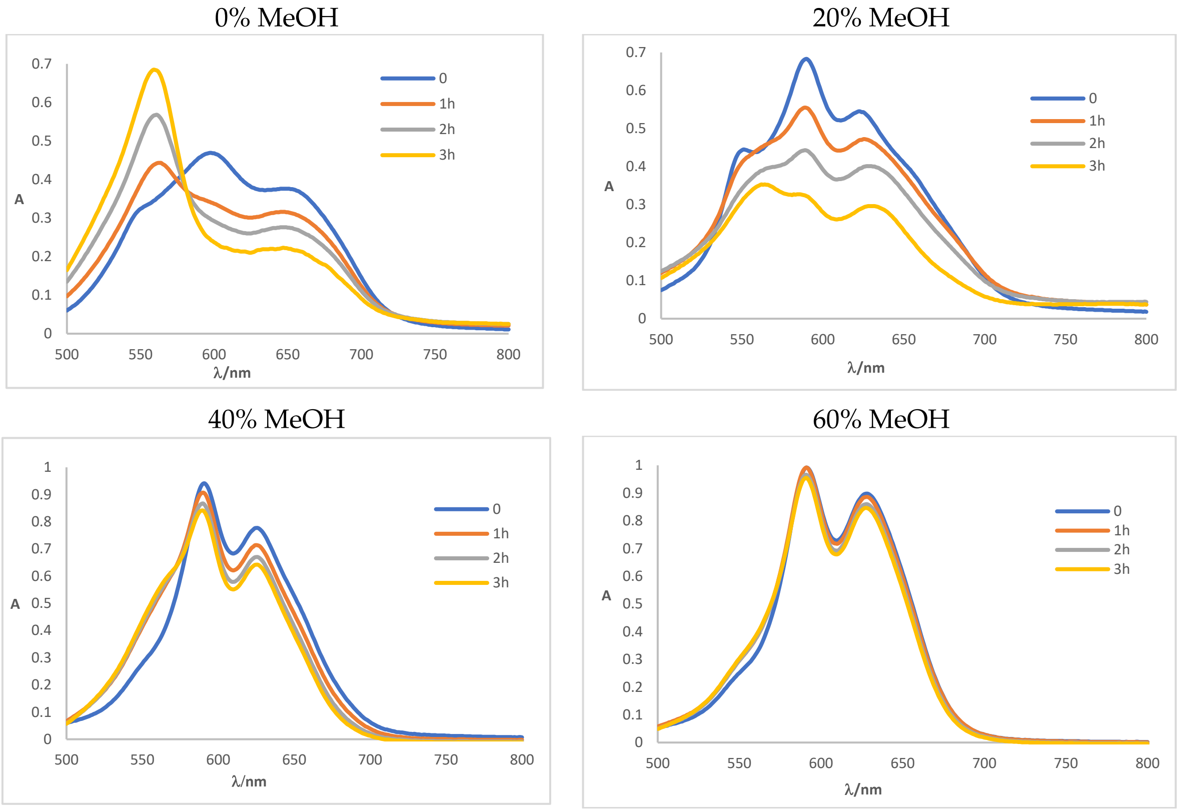

Figure S4: Absorption spectra of Bis-PMS **5** (5.5 μM) after incubation points in EMEM with and without MeOH (0, 20, 40 and 60%; *v*/*v*)

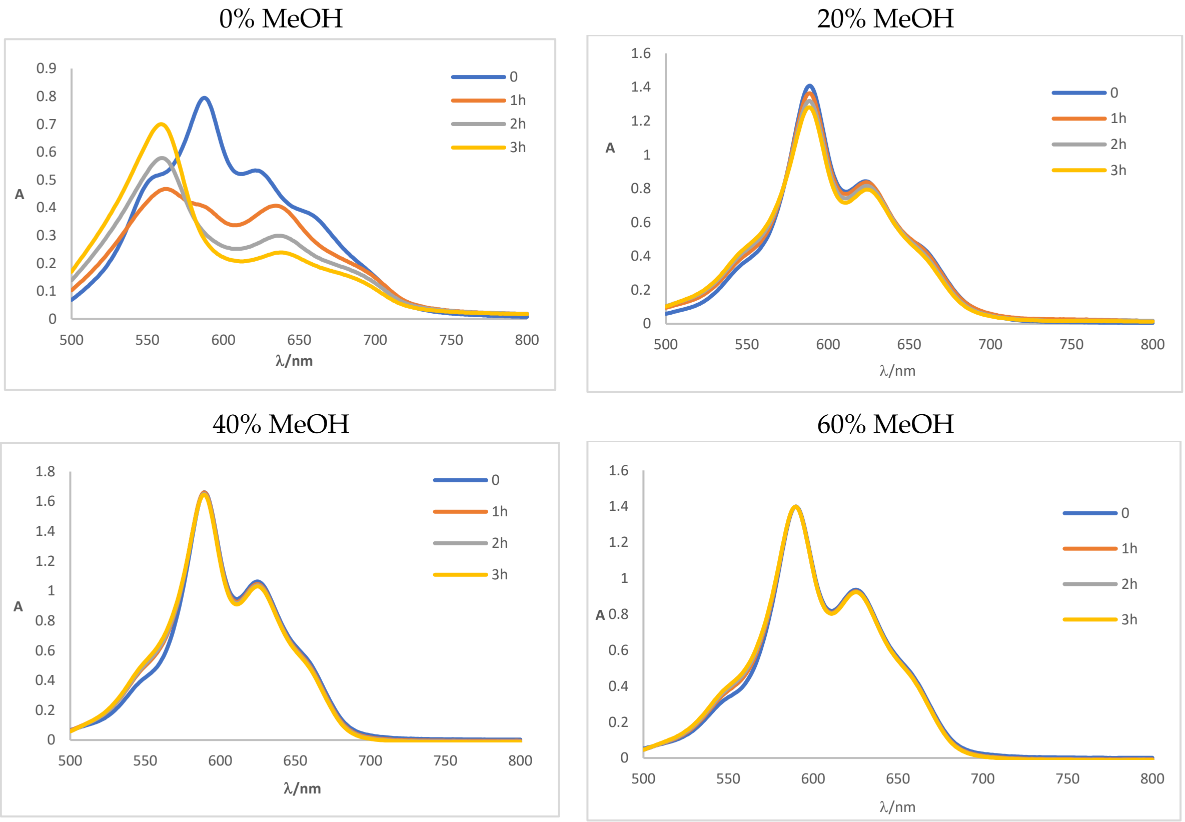

Figure S5: Absorption spectra of Bis-PMS **6** (**5.5** μM) after incubation in EMEM with and without MeOH (0, 20, 40 and 60%; *v*/*v*)

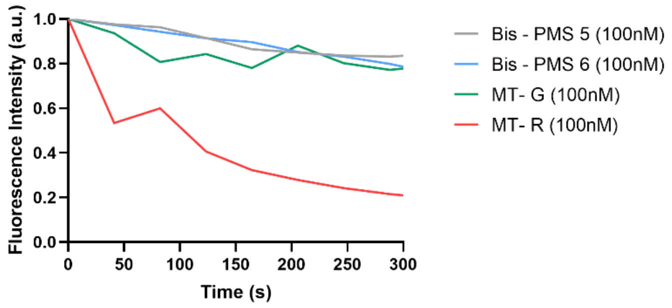

Figure S6: Normalized bleaching curves of Bis-PMS **5** and **6**, MitoTracker™ Green FM (MT-G), and MitoTracker™ Red FM (MT-R). Fluorescence intensity was measured for 5 minutes in BJ-hTERT cells using a Leica TCS SP8 WLL SMD-FLIM confocal microscope with an HC PL APO CS2 63x / 1.2W water immersion objective. For Bis-PMS **5** and **6, t**he excitation wavelengths were 630 nm and the emission was recorded in the 650–670 nm range. For MT-G, the excitation wavelength was 490 nm and the emission was measured in the 510–550 nm range. MT-R was excited at 581 nm and the emission was recorded in the 640–650 nm range.

Docking studies

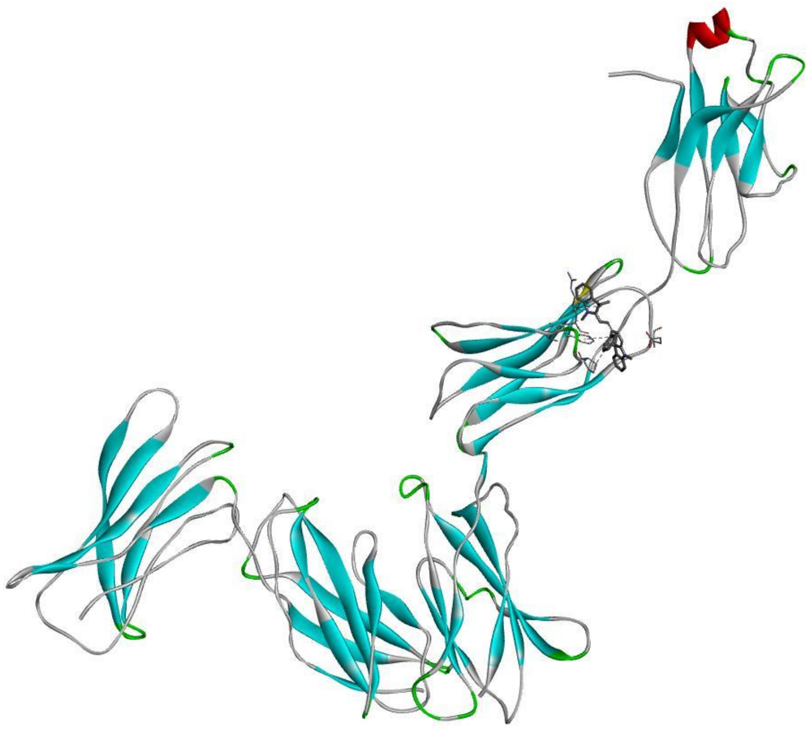

Figure S7: Interaction of a mono-penhamethinium salt with a 3D model of IL-6R. The binding site (cavity) is localized in IL-6Rβ.

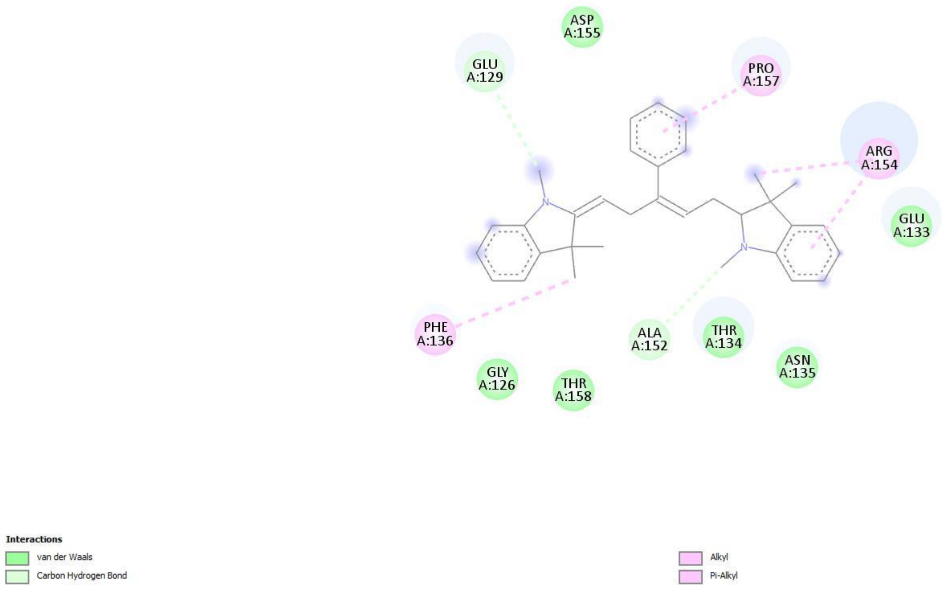

Figure S8: Interaction of a mono-pentamethinium salt with the residues of homology model of IL-6R.

In vitro studies of bispentamethinum salts 5 and 6

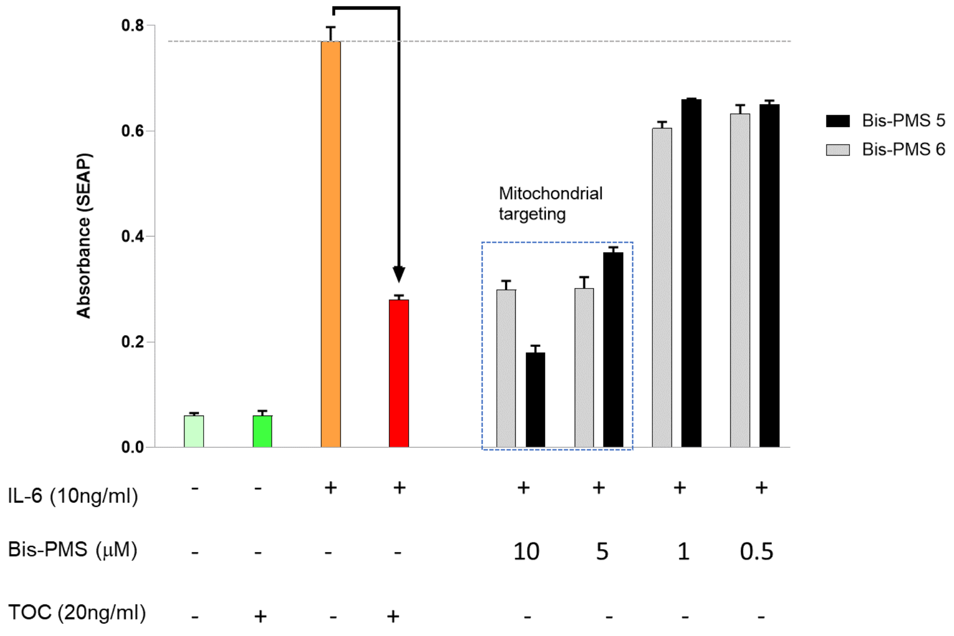

Figure S9: Effect of bis-pentamethinum salts **5** and **6** on the IL-6 signaling cascade. HEK-Blue™ IL-6 reporter cells were used for a secreted embryonic alkaline phosphatase (SEAP) assay to evaluate IL-6 mediated signaling. The selective IL-6R inhibitor tocilizumab (TOC) extensively inhibited STAT3-depedent production of alkaline phosphatase (AP) after stimulation of the cells by exogenous IL-6 for 24 hours. Reduction of AP production by 10 and 5 µM Bis-PMS **5** and **6** is most likely caused by their inhibition of mitochondrial respiration. Low concentrations (1 and 0.5 µM) of Bis-PMS **5** and **6** were not toxic for the cells in this experimental setup and mildly inhibited AP production. Data are presented as mean ± SD.

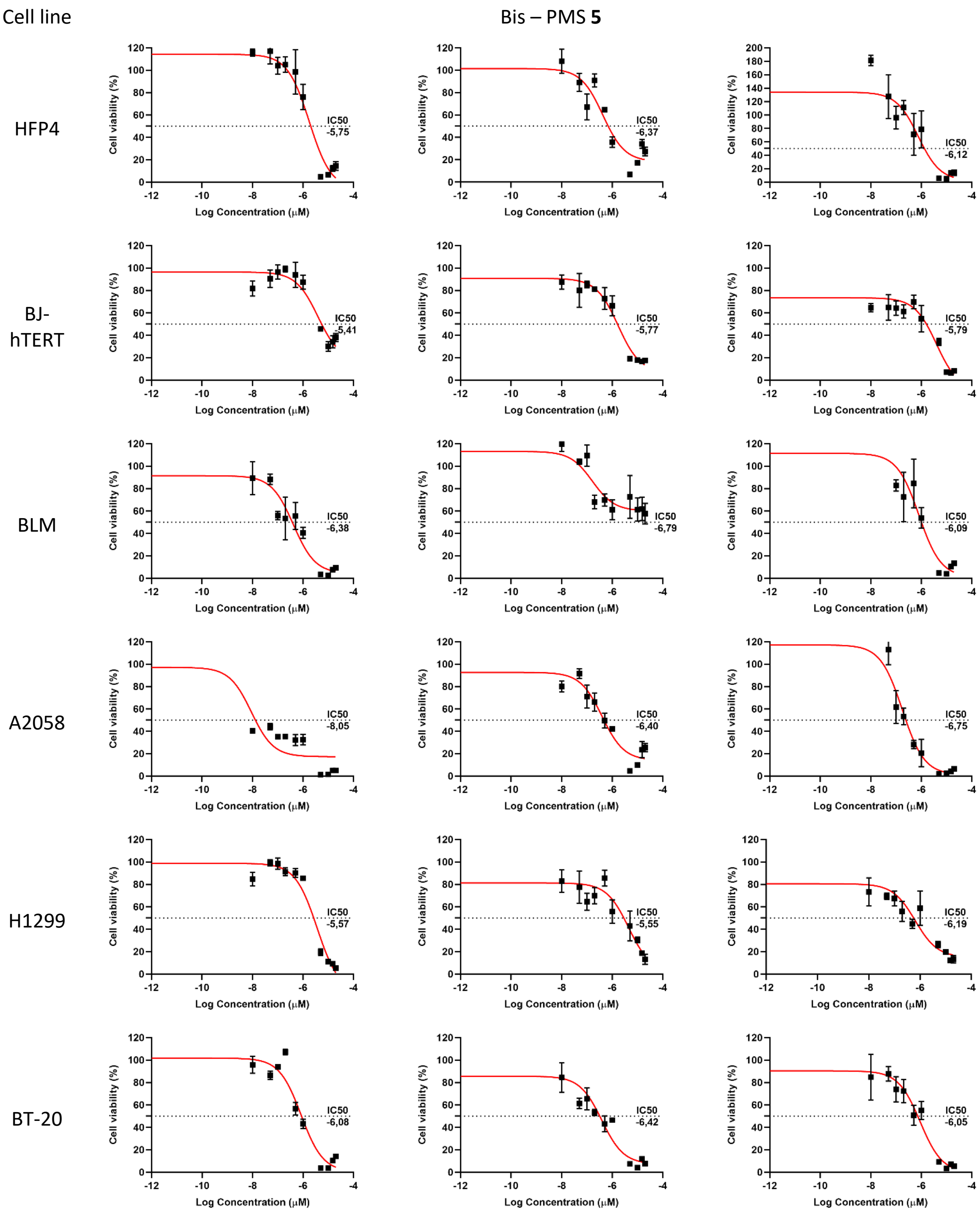
Cell line
Bis – PMS 5
HFP4
IC50 -5,75
IC50 -6,37
IC50 -6,12
BJ-hTERT
IC50 -5,41
IC50 -5,77
IC50 -5,79
BLM
IC50 -6,38
IC50 -6,79
IC50 -6,09
A2058
IC50 -8,05
IC50 -6,40
IC50 -6,75
H1299
IC50 -5,57
IC50 -5,55
IC50 -6,19
BT-20
IC50 -6,08
IC50 -6,42
IC50 -6,05
Cell viability (%)
Log Concentration (μM)

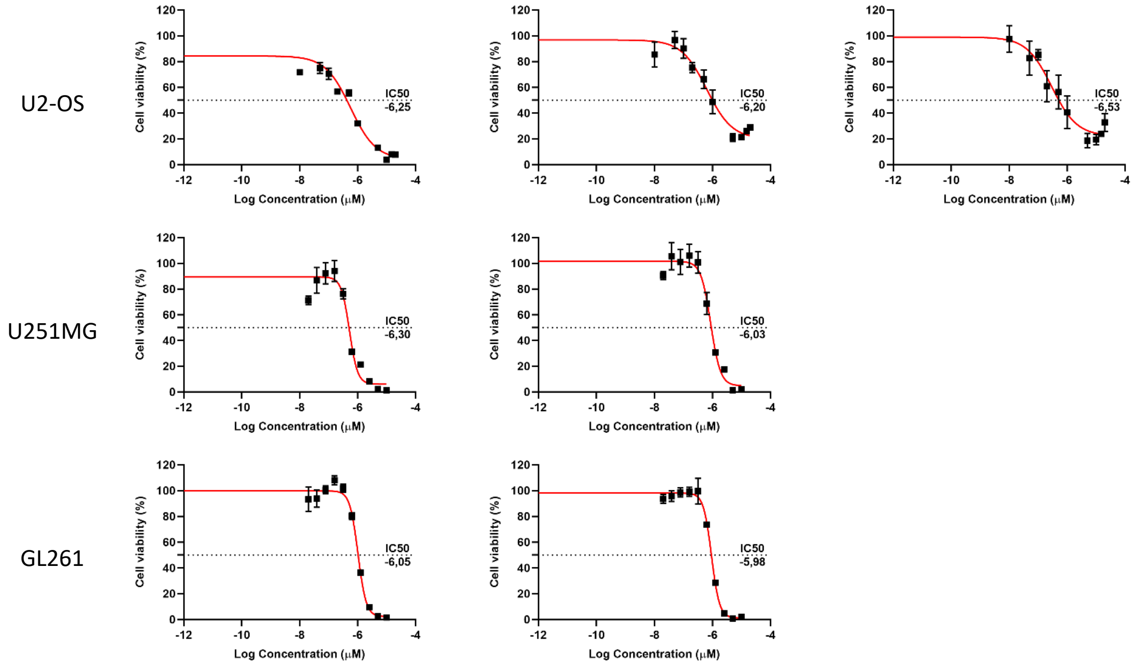

Figure **S10**: Cytotoxity of Bis-PMS **5** in normal ((HF P4 and BJ-hTERT human fibroblasts) and cancer cells ((BLM and A2058 human melanoma cells, H1299 human non-small cell lung cancer cells, human BT-20 breast cancer cells, human U251MG and mouse Gl261 glioblastoma cells, and U2-OS human osteosarcoma cells).

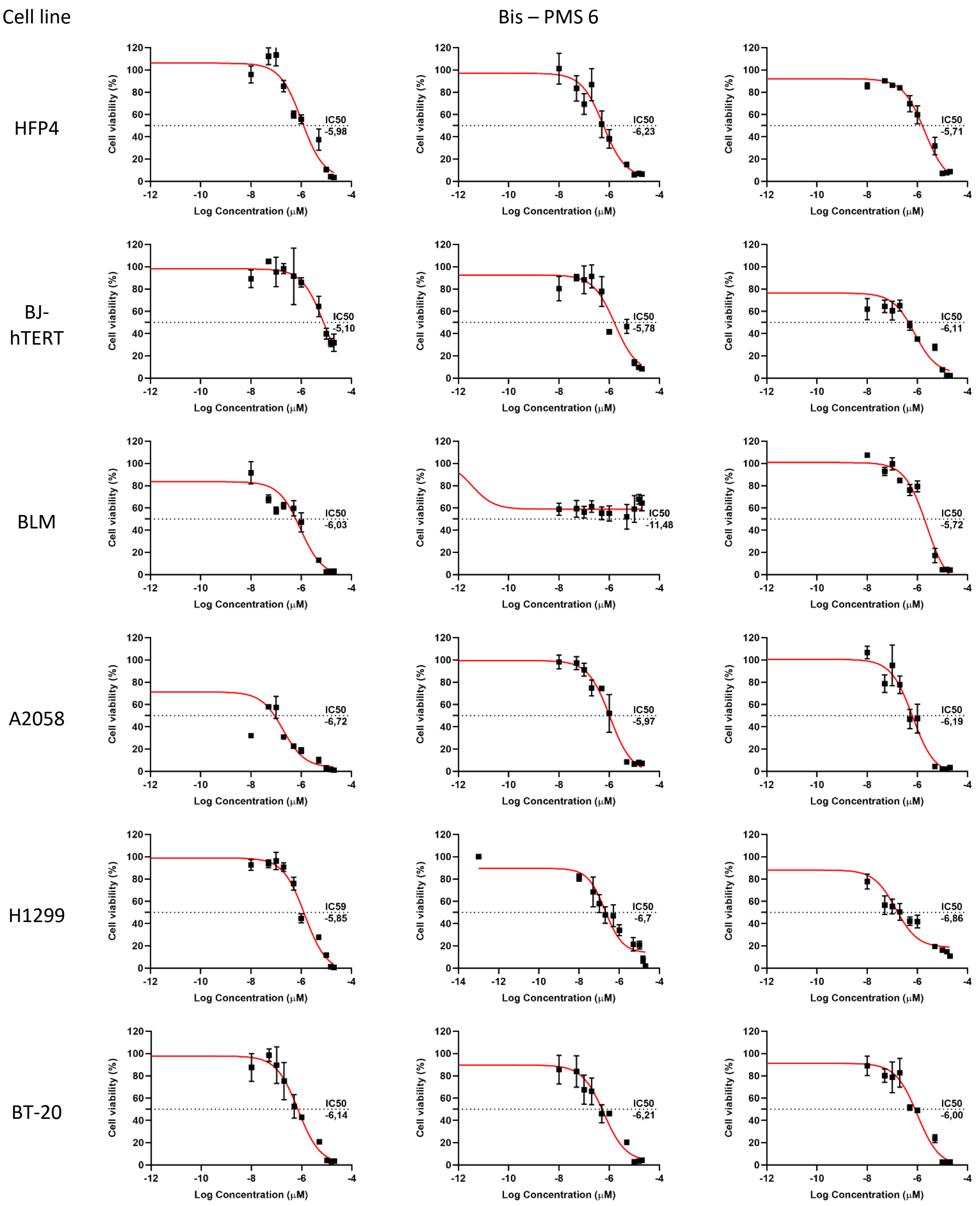
Cell line
Bis – PMS 6
HFP4
BJ-hTERT
BLM
A2058
H1299
BT-20
Cell viability (%)
Log Concentration (µM)
IC50 -5,98
IC50 -6,23
IC50 -5,71
IC50 -5,10
IC50 -5,78
IC50 -6,11
IC50 -6,03
IC50 -11,48
IC50 -5,72
IC50 -6,72
IC50 -5,97
IC50 -6,19
IC59 -5,85
IC50 -6,7
IC50 -6,86
IC50 -6,14
IC50 -6,21
IC50 -6,00

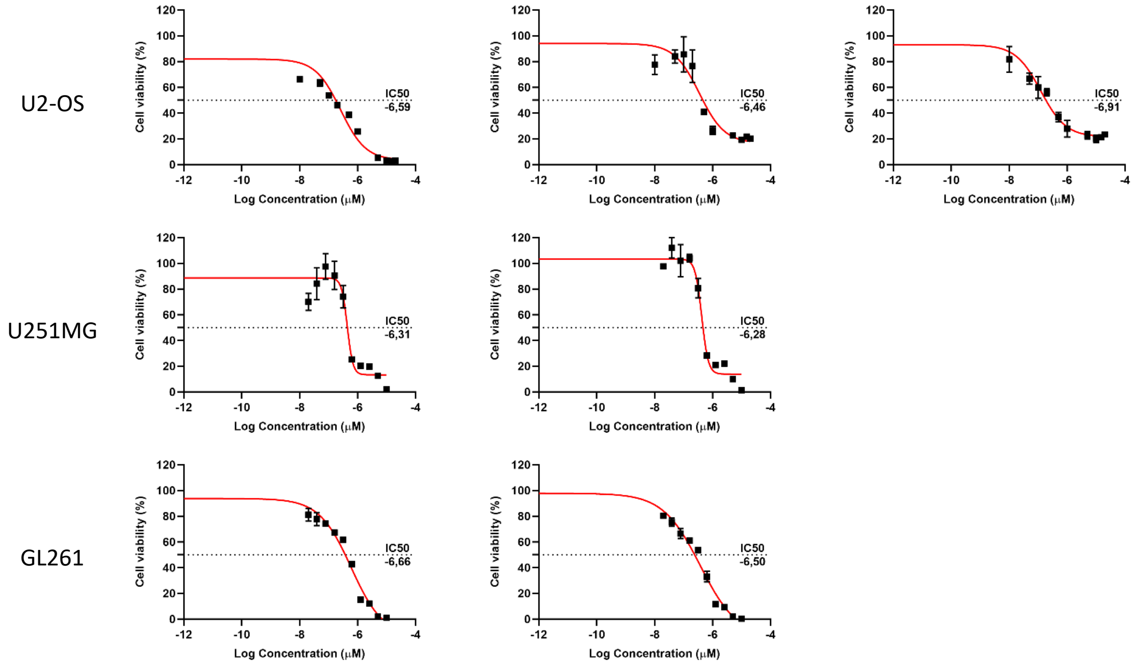

Figure S11: Cytotoxity of Bis-PMS **6** in normal ((HF P4 and BJ-hTERT human fibroblasts) and cancer cells ((BLM and A2058 human melanoma cells, H1299 human non-small cell lung cancer cells, human BT-20 breast cancer cells, human U251MG and mouse Gl261 glioblastoma cells, and U2-OS human osteosarcoma cells).

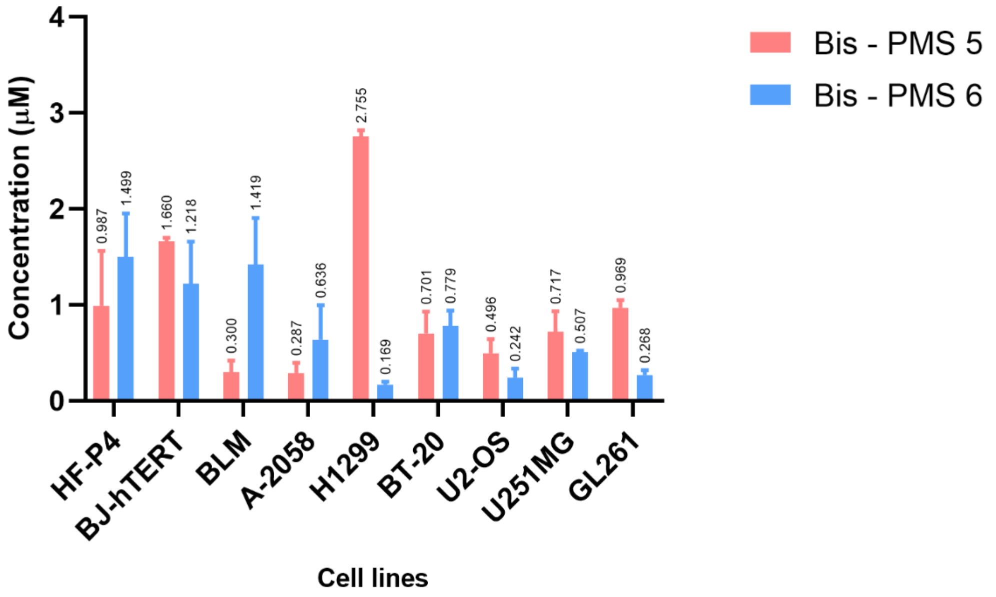

Figure S12: Cytotoxity of Bis-PMS **5** and **6** in normal ((HF P4 and BJ-hTERT human fibroblasts) and cancer cells ((BLM and A2058 human melanoma cells, H1299 human non-small cell lung cancer cells, human BT-20 breast cancer cells, human U251MG and mouse Gl261 glioblastoma cells, and U2-OS human osteosarcoma cells).

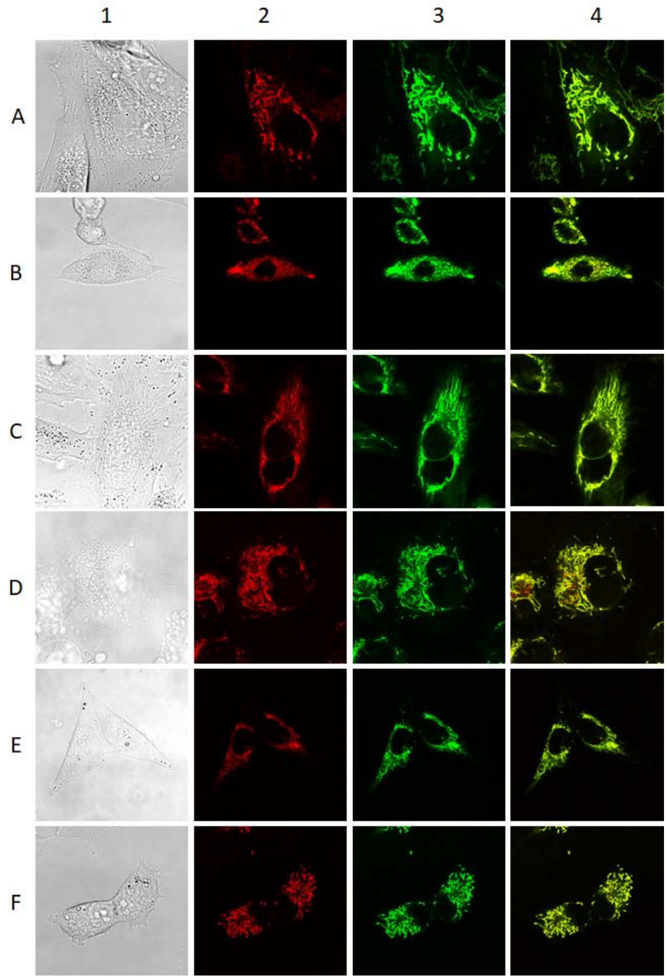

Figure S13: In vitro localization of Bis-PMS **5** in BJ-hTERT (**A**), HF-P4 (**B**), A-2058 (**C**), BLM (**D**), U-2 OS (**E**), and H1299 (**F**) cells. All cell samples were stained with MT-G, 300 nM (green) and Bis-PMS **5**, 100 nM (red), where (1) bright-field image, (2) fluorescence imaging of Bis-PMS **5**, (3) fluorescence imaging of MT-G, and (4) overlay of (2) and (3). Images represent microscopy results from at least six independent experiments. Red channel: λex = 630 nm, λem = 650–670 nm; green channel: λex = 490 nm, λem= 510–516 nm.

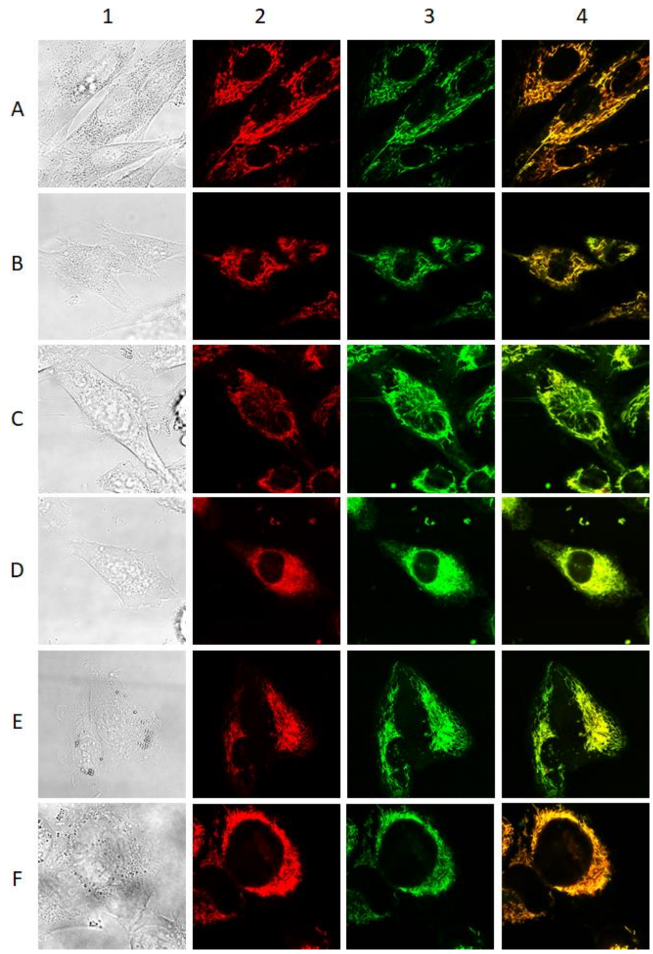

Figure S14: In vitro localization of Bis-PMS **6** in BJ-hTERT (**A**), HF-P4 (**B**), A-2058 (**C**), BLM (**D**), U-2 OS (**E**), and H1299 (**F**) cells. All cell samples were stained with MT-G, 300 nM (green) and bis-pentamethinium salt **6**, 100 nM (red), where (1) bright-field image, (2) fluorescence imaging of Bis-PMS, (3) fluorescence imaging of MT-G, and (4) overlay of (2) and (3). Images represent microscopy results from at least six independent experiments. Red channel: λex= 630 nm, λem= 650–670 nm; green channel: λex= 490 nm, λem= 510–516 nm

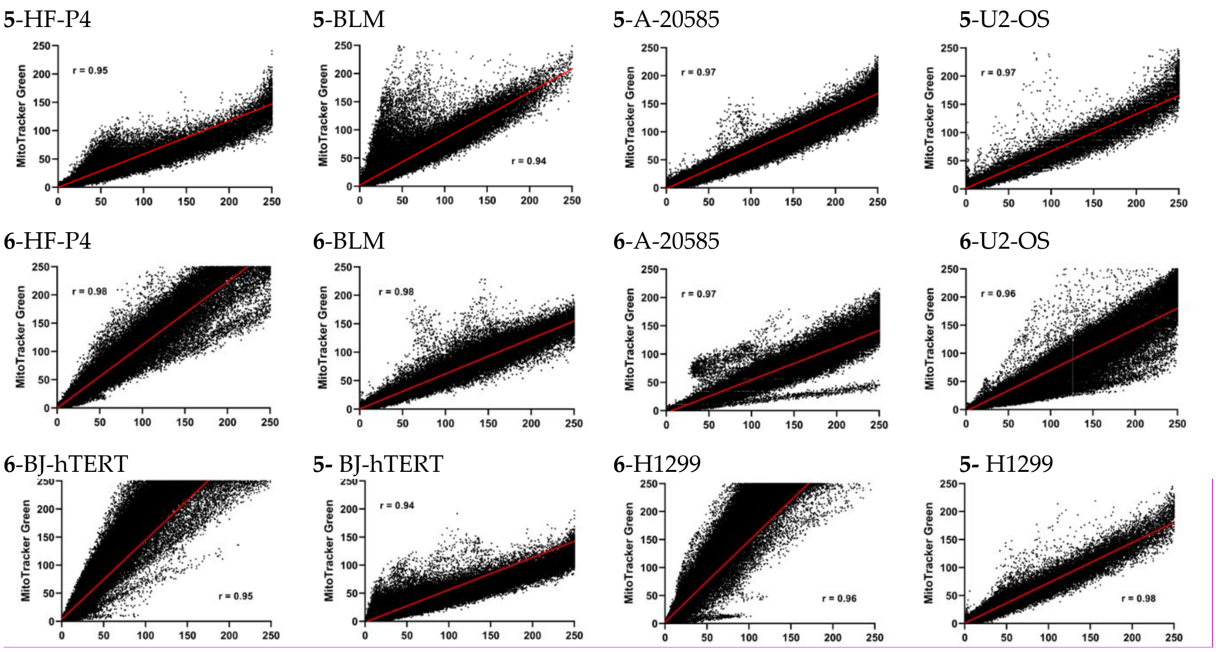

Figure S15: 2-D scatter plots of fluorescence of Bis-PMS **5** and **6** versus MitoTracker Green (MT-G) (in merged images from S6 and S7) with linear regression. Pearson's correlation coefficient (r)

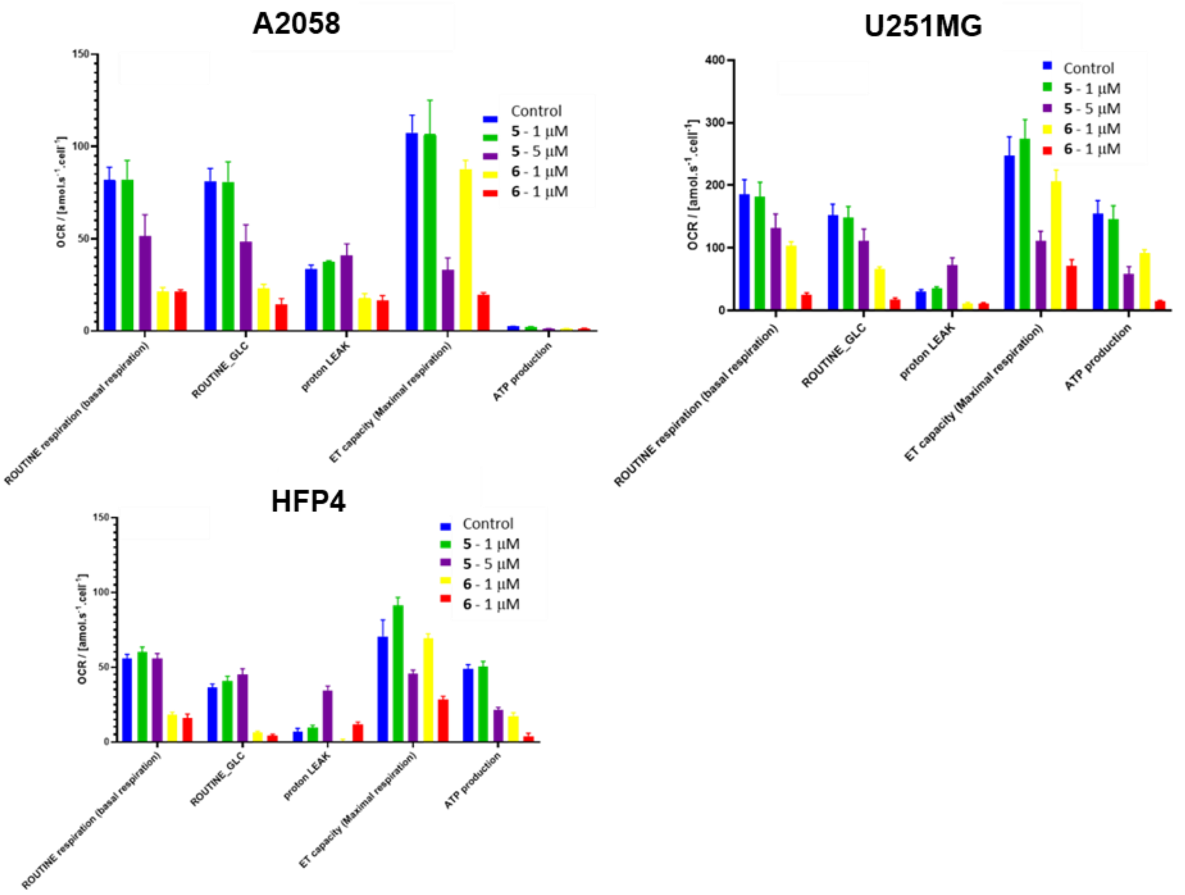

Figure S16: Influence of Bis-PMS **5** and **6** on mitochondrial metabolism and respiration in A2058, U251MG and HFP4 cells. OCR = oxygen consumption rate

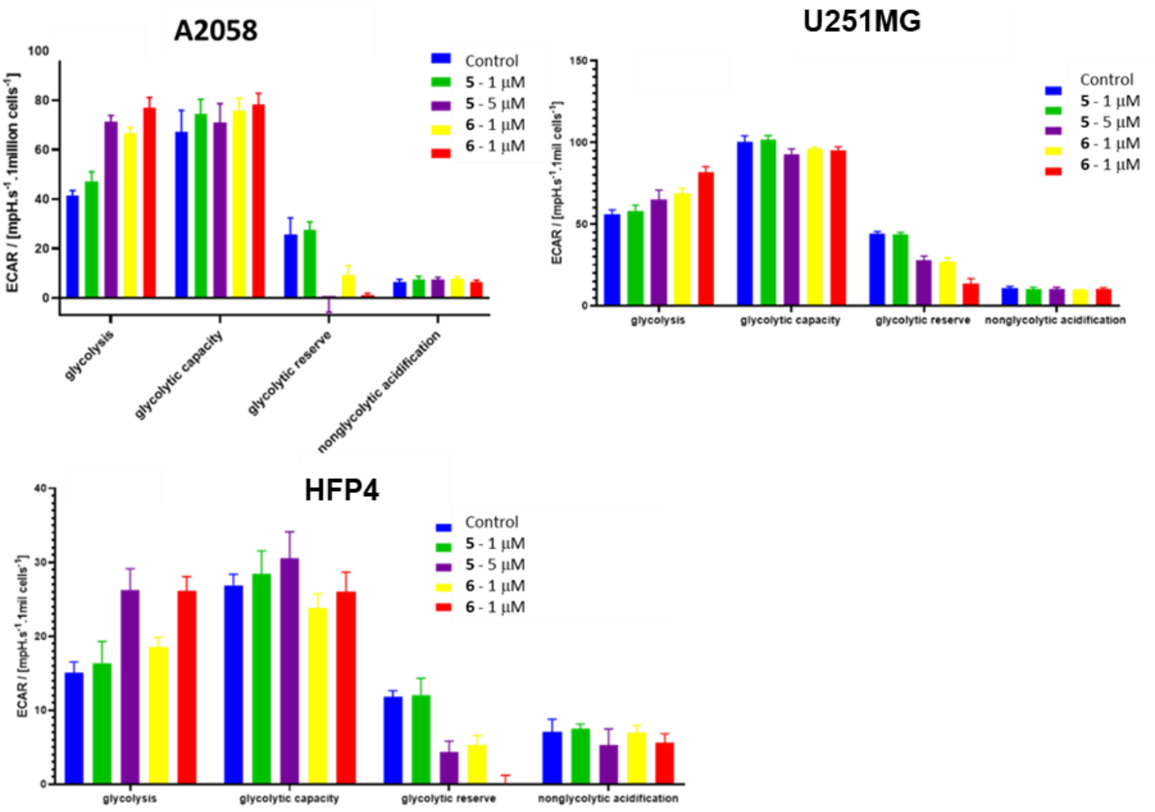

Figure S17: Influence of Bis-PMS **5** and **6** on glycolytic function in A2058, U251MG and HFP4 cells. ECAR = extracellular acidification rate

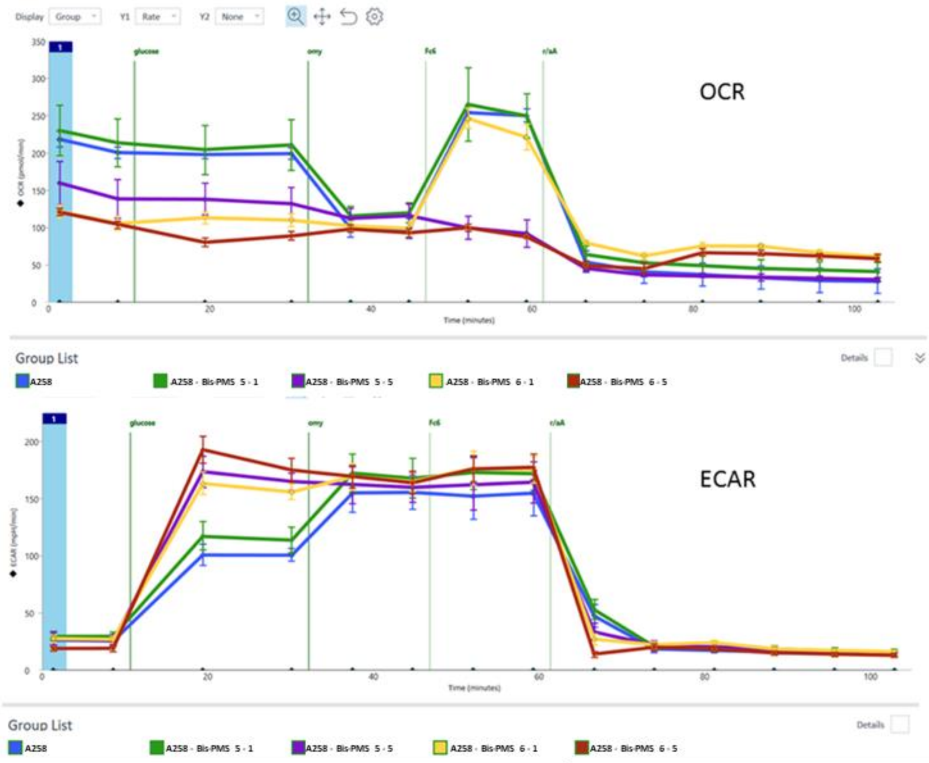

Figure S18: Measurements of respiration (OCR) and glycolysis (ECAR) in A2058 melanoma cells. The inhibitory effect of Bis-PMS **5** and **6** on mitochondrial respiration and stimulation of glycolysis is well visible.

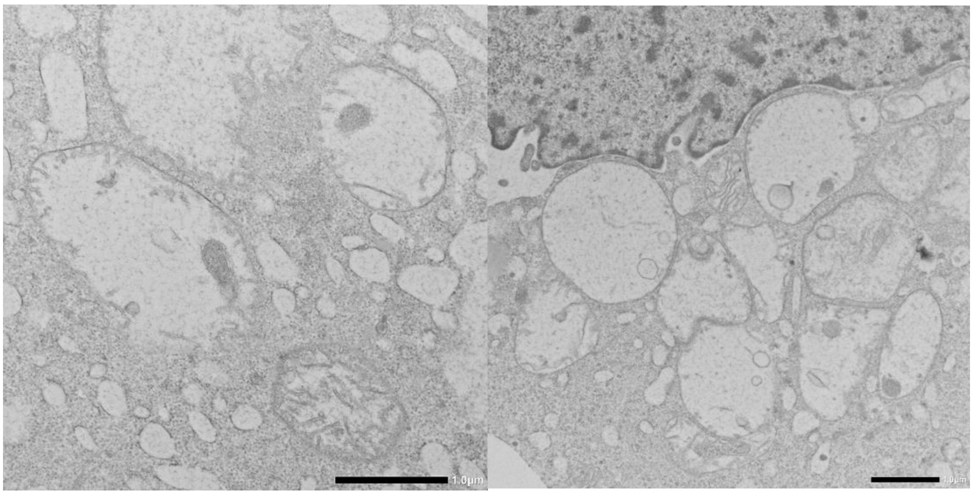

Figure S19: Influence of Bis-PMS **5** on mitochondrial morphology of A2058 cells. Mitochondria can be seen in various stages of degradation. Some still contain numerous cristae, have normal size, and their membrane appears intact; others are abnormally enlarged and their cristae with both membranes are degraded. Bar is 1 μm.

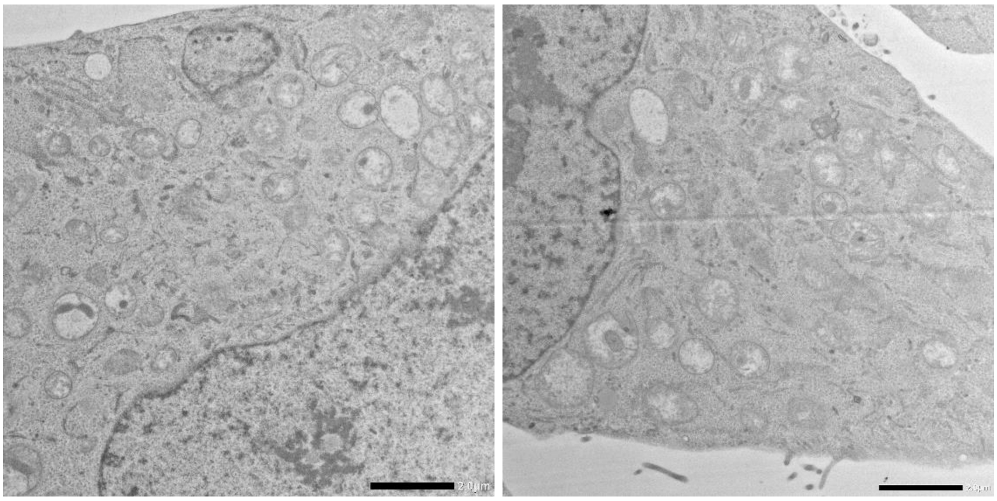

Figure S20: Influence of Bis-PMS **6** on mitochondrial morphology in A2058 cells. High magnification images show that the majority of mitochondria have abnormal circular shape, even though their size is significantly smaller in comparison to cells treated with Bis-PMS **5**.

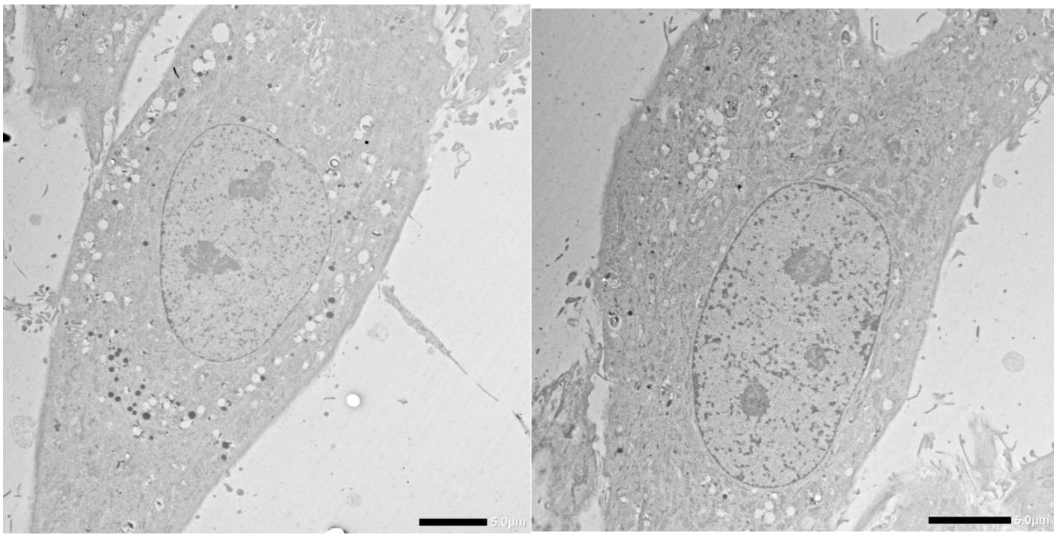

Figure S21: Low magnification overview of control HFP4 cells. Autolytic vacuoles are abundant, but relatively small in comparison to cells treated with Bis-PMS **5** and **6**. Most of the cytoplasm is filled with typical mitochondria and rough ER, lipid droplets are also present. Bar is 5 μm.

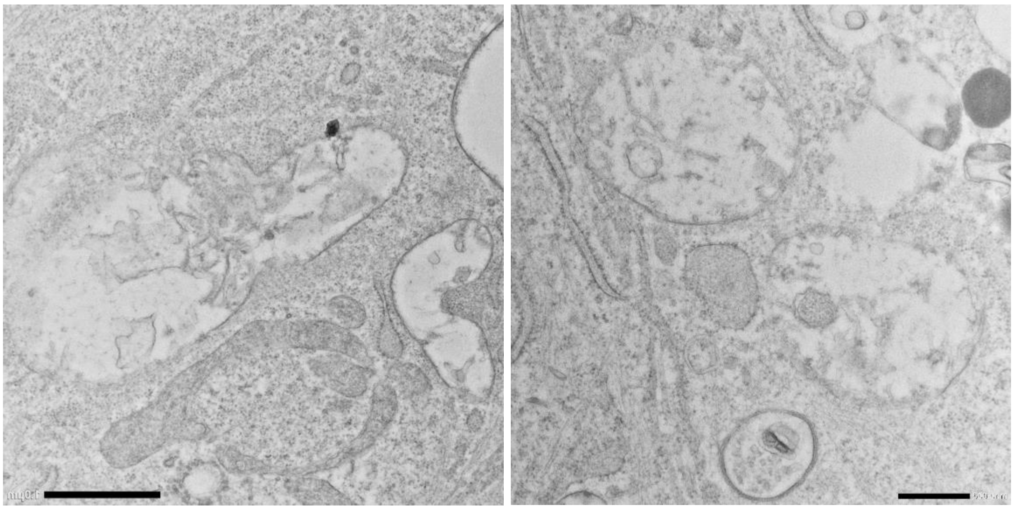

Figure S22: Influence of Bis-PMS **5** on mitochondrial morphology in HFP4 cells. Early stages of mitochondrial degradation. Previously dense matrix turns into clear with a web-like structure, the organized system of cristae is degraded into disarranged bulks of membrane components with only few peripheral cristae retaining their structure. Mitochondria can be distinguished from autolytic vacuoles by the presence of a double membrane. Bar is 1 μm (**left**) and 0.5 μm (**right**).

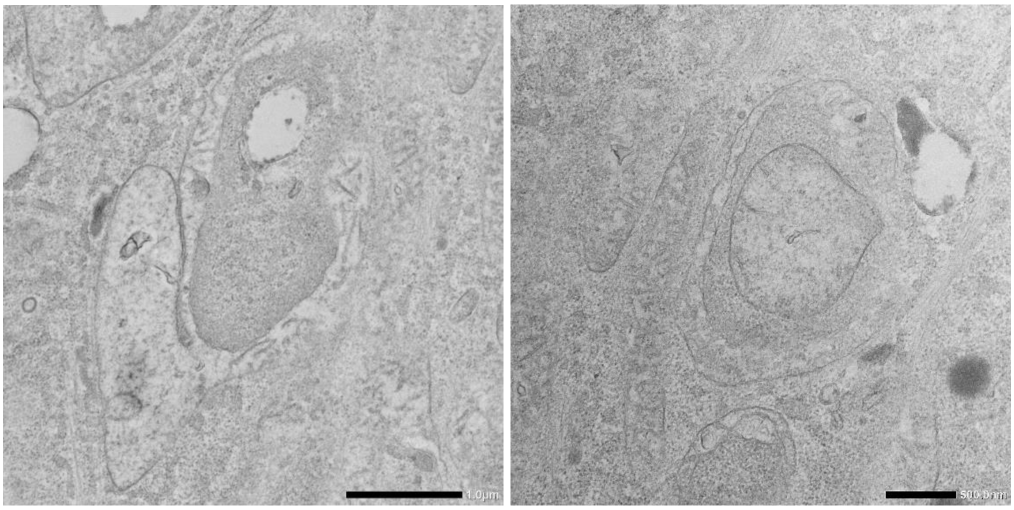

Figure S**23**: Influence of Bis-PMS **6** on mitochondrial morphology in HFP4 cells. The majority of mitochondria have the shape and size comparable to controls, but their matrix is not so dense and the system of cristae displays morphological deformations. Bar is 1 μm and 0.5 μm (right).

Table S1: Excitation and emission maxima of Bis-pentamethinium salts **5** and **6**

**Salt**	**Solvent**	**Excitation maxima (nm)**	**Emission maxima (nm)**
Bis-PMS **5**	DMSO	644	677
	EtOH	639	663
	MeOH	637	661
	PBS	637	655
Bis-PMS **6**	DMSO	642	665
	EtOH	639	662
	MeOH	634	659

	PBS	637	655

Table S2: Interaction mode of Bis-pentamethinium salts **5** and **6** with amino acid residues of IL-6Rβ

A chain	Bis-PMS 5	Bis-PMS 6
Phe69	π-alkyl	π-π
Ile52	alkyl	π-alkyl
Ile77	alkyl	alkyl and π-alkyl
Ala73	Alkyl and π-alkyl	π-alkyl
Phe50	π-π and π-alkyl	π-π and π-sigma
Asn48	Alkyl	-
His49	π donor hydrogen bond	-
Pro53	-	π-alkyl
Gln56	van der Waals	van der Waals
Thr70	van der Waals	-
Asp71	van der Waals	-
Ile72	van der Waals	-
Asn48	van der Waals	van der Waals
Ser74	π donor hydrogen bond	van der Waals
Thr47	-	van der Waals
Asn76	van der Waals	van der Waals

Table S3. Cytotoxicity of Bis-PMS **5** and **6** in normal and cancer cells. IC_{50} (µmol/L) of Bis-PMS **5** and **6** was determined in HF-P4 and BJ-hTERT human fibroblasts, BLM and A2058 human melanoma cells, H1299 human non-small cell lung cancer cells, human BT-20 breast cancer cells, human U251MG and mouse Gl261 glioblastoma cells, and U2-OS human osteosarcoma cells.

Cell lines	Bis-PMS 5 (IC_{50} / µM)			Bis-PMS 6 (IC_{50} / µM)		
	1.	2.	3.	1.	2.	3.
HFP4	1.778	0.427	0.757	1.047	0.589*	1.95
BJ-hTERT	3.89*	1.698	1.622	7.94*	1.66	0.776
BLM	0.417	0.162*	0.183	0.933	0*	1.905
A2058	0.00891*	0.396	0.178	0.191	1.072	0.646
H1299	2.692	2.818	0.646*	1.38*	0.199	0.138
BT-20	0.832	0.38	0.891	0.724	0.617	0.997
U2-OS	0.562	0.631	0.295	0.257	0.347	0.123
U251	0.501	0.938	-	0.485	0.526	-
Gl261	0.900	1.043	-	0.218	0.319	-

* excluded values
